# Internal and external bidirectional traction facilitating endoscopic submucosal excavation of a huge subepithelial tumor in the fornix of the gastric fundus near the cardia

**DOI:** 10.1055/a-2291-9183

**Published:** 2024-04-11

**Authors:** Zhi-qiang Du, Jing Tang, Wei-hui Liu

**Affiliations:** 1 546231 Department of Gastroenterology, The Peopleʼs Hospital of Jianyang City, Jianyang, China; 2 159414 Department of Gastroenterology, Guangyuan Central Hospital, Guangyuan, China; 3 Department of Gastroenterology and Hepatology, Sichuan Provincial Peopleʼs Hospital, School of Medicine, Chengdu, China

Endoscopic submucosal excavation (ESE) and endoscopic full-thickness resection (EFTR) are efficacious and reliable methods for managing gastrointestinal subepithelial tumors 1 2 3 . Some traction methods are often used to expose subepithelial tumors 3 . However, traditional traction methods may be inadequate for full exposure and complete excavation 4 5 . To address this issue, we have developed an internal and external bidirectional traction method that has been successfully utilized in ESE ( Video 1 ).

Endoscopic submucosal excavation of a huge subepithelial tumor in the fornix of the gastric fundus near the cardia using internal and external bidirectional traction.Video 1

A 40-year-old man with a huge subepithelial tumor located in the fornix of the gastric fundus near the cardia was referred for ESE treatment ( Fig. 1 **a** ). Due to the specific location and the limited angle of the endoscope, it was difficult to get close to the tumor. First, a clip with a rubber band was attached to the mucosa on the anal side of the tumor. Then the second clip picked up the rubber band and fixed it to the greater curvature mucosa opposite the gastric fundus ( Fig. 1 **b** ). After transverse incision of the mucosa, the tumor was clearly exposed ( Fig. 1 **c** ). We dissected the outer edge of the tumor and found that it was difficult to expose the boundary. The third clip with dental floss was fixed on the oral side of the incised mucosa ( Fig. 1 **d** ). By pulling the dental floss and using air control, the boundary of the tumor was clearly exposed. Under lifting force of the internal and external bidirectional traction, the tumor was excavated quickly and completely ( Fig. 1 **e** ). Since the surgical wound was well presented using this traction method, we performed sufficient hemostasis on the entire wound ( Fig. 1 **f** ).

**Fig. 1:**
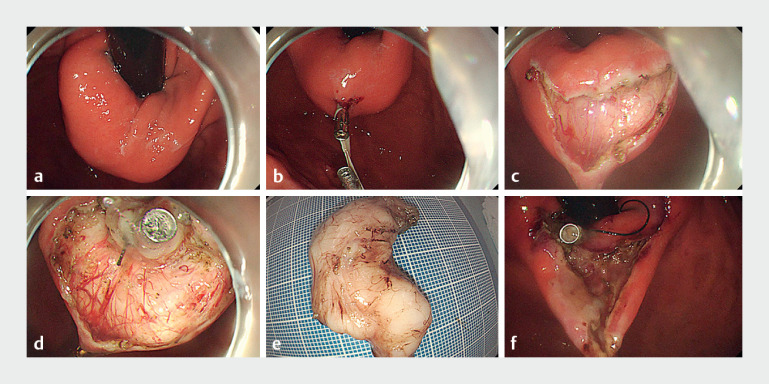
Schematic picture of the internal and external bidirectional traction method for endoscopic submucosal excavation of a huge subepithelial tumor in the fornix of gastric fundus near the cardia. **a** A subepithelial tumor measuring about 5.0 × 3.0 cm in the fornix of gastric fundus near the cardia. **b** A clip with a rubber band was attached to the mucosa on the anal side of the lesion and the second clip picked up the rubber band and was fixed on the mucosa of the greater curvature opposite to the gastric fundus. **c** Transverse incision of the mucosa in the middle of the tumor and clear exposure of the tumor under internal traction. **d** The third clip with dental floss was fixed on the oral side of the incised mucosa. **e** The tumor was dissected with the complete capsule. **f** Sufficient hemostasis on the surgical wound was performed with the help of internal and external bidirectional traction.

This method helps to expose the tumor quickly during incision of the mucosa, meanwhile ensuring dissection of the tumor with the complete capsule. It does not require special instruments and may be a suitable method for the ESE and EFTR in some special sites.

Endoscopy_UCTN_Code_TTT_1AO_2AG

## Notes

Endoscopy E-Videos https://eref.thieme.de/e-videos
*E-Videos* is an open access online section of the journal *Endoscopy* , reporting on interesting cases and new techniques in gastroenterological endoscopy. All papers include a high-quality video and are published with a Creative Commons CC-BY license. Endoscopy E-Videos qualify for HINARI discounts and waivers and eligibility is automatically checked during the submission process. We grant 100% waivers to articles whose corresponding authors are based in Group A countries and 50% waivers to those who are based in Group B countries as classified by Research4Life (see: https://www.research4life.org/access/eligibility/ ). This section has its own submission website at https://mc.manuscriptcentral.com/e-videos .
